# Anomaly Detection for Asynchronous Multivariate Time Series of Nuclear Power Plants Using a Temporal-Spatial Transformer

**DOI:** 10.3390/s24092845

**Published:** 2024-04-29

**Authors:** Shuang Yi, Sheng Zheng, Senquan Yang, Guangrong Zhou, Jiajun Cai

**Affiliations:** 1 College of Electrical Engineering and New Energy, China Three Gorges University, Yichang 443002, China; yishuang@ctgu.edu.cn (S.Y.); cjj_ctgu@ctgu.edu.cn (J.C.); 2 College of Science, China Three Gorges University, Yichang 443002, China; zhougr0423@ctgu.edu.cn; 3 China Nuclear Power Operation Technology Corporation, Ltd., Wuhan 430074, China; yangsq@cnnp.com.cn; 4 China Nuclear Industry Key Laboratory of Simulation Technology, Wuhan 430074, China

**Keywords:** anomaly detection, temporal-spatial transformer, asynchronous multivariate time series, nuclear power plants

## Abstract

Industrial process monitoring is a critical application of multivariate time-series (MTS) anomaly detection, especially crucial for safety-critical systems such as nuclear power plants (NPPs). However, some current data-driven process monitoring approaches may not fully capitalize on the temporal-spatial correlations inherent in operational MTS data. Particularly, asynchronous time-lagged correlations may exist among variables in actual NPPs, which further complicates this challenge. In this work, a reconstruction-based MTS anomaly detection approach based on a temporal-spatial transformer is proposed. It employs a two-stage temporal-spatial attention mechanism combined with a multi-scale strategy to learn the dependencies within normal operational data at various scales, thereby facilitating the extraction of temporal-spatial correlations from asynchronous MTS. Experiments on simulated datasets and real NPP datasets demonstrate that the proposed model possesses stronger feature learning capabilities, as evidenced by its improved performance in signal reconstruction and anomaly detection for asynchronous MTS data. Moreover, the proposed TS-Trans model enables earlier detection of anomalous events, which holds significant importance for enhancing operational safety and reducing potential losses in NPPs.

## 1. Introduction

Nuclear power, recognized as a clean and sustainable energy source, plays a crucial role in addressing climate change and achieving carbon neutrality [1,2]. However, concerns over potential safety risks have limited the wider expansion of nuclear power [3,4]. As a crucial measure in routine maintenance, accurate and efficient operational condition monitoring can detect anomalous events at an early stage and supply essential information for subsequent maintenance actions and fault diagnosis [5,6]. This is particularly significant for enhancing operational safety and minimizing or averting losses in safety-critical systems such as NPPs.

With the advent of the Internet of Things (IoT) and advanced Instrumentation and Control (I&C) technologies, a wide array of sensors have been deployed to gather real-time operational data from NPPs. Over the years, these continual data streams have culminated in the creation of a comprehensive MTS database. This database is highly conducive to leveraging data-driven MTS anomaly detection techniques for condition monitoring in NPPs [7]. The MTS data, sourced from diverse sensor observations, are subject to temporal fluctuations and exhibit pronounced cross-channel coupling due to intrinsic mechanistic interrelations. The application of deep learning, celebrated for its remarkable prowess in feature dimensionality reduction and extraction [8,9], to anomaly detection of complex, nonlinear, and intricately coupled systems like NPPs, is imbued with significant potential [10]. Nonetheless, there exist several challenges that necessitate comprehensive consideration and resolution.

One of the primary limitations of using deep learning methods for anomaly detection of MTS data is that many of these methods fail to fully leverage the temporal-spatial correlations inherent in MTS. Some of these approaches favor exploiting the spatial correlation features among the variables of MTS to facilitate anomaly detection. They utilize neural networks such as CNN, GNN, and AE to capture the cross-channel spatial correlations within the MTS [11,12,13]. These spatial correlations can be recognized as the associations among different sensor variables. An anomaly is considered to have occurred when these associations deviate from the normal pattern. However, similar to statistics-based approaches such as PCA, these methods neglect the temporal dependencies of MTS, which may result in the inability to detect certain types of anomalies, such as contextual anomalies.

Certain other approaches place a greater emphasis on capturing temporal dependencies. They employ networks such as Temporal Convolutional Networks (TCNs), Recurrent Neural Networks (RNNs), Long Short-Term Memory (LSTM), or transformers to capture the sequential correlations across various time steps [14,15,16,17]. Notably, the transformer model [18], with its efficient parallel processing capabilities to handle long-range temporal dependencies and its robust capacity for modeling sequential data, has achieved remarkable success in fields such as Natural Language Processing (NLP), Computer Vision (CV), and speech processing [19]. However, for analyzing strongly coupled MTS, the vanilla transformer, may not fully leverage the inter-variable spatial correlations [20]. This may be attributed to the fact that the vanilla transformer model treats multivariate observations at a time step as an embedding and then employs a self-attention mechanism to establish connections across different time steps. However, this approach does not sufficiently explore the correlations between variables. Particularly in real-world scenarios like nuclear power plants, MTS data are collected from various sensors that may exhibit time-delay correlations (asynchronous correlations) [21] owing to disparities in sensor locations, noise levels, and parameter response times. Under such circumstances, models that utilize point-wise embedding based on synchronous timestamps may struggle to accurately capture the correct cross-channel spatial correlations. This shortcoming becomes even more pronounced in states of transient conditions where data are sparse.

In this paper, we introduce a temporal-spatial transformer-based model designed for anomaly detection of nuclear power operational data. The proposed model utilizes a channel-independent patch-wise embedding technique to achieve embedding representations of the input data. A two-stage temporal-spatial attention mechanism is employed to capture more extensive temporal dependencies and cross-channel spatial correlations within the MTS data. In this two-stage attention framework, the output derived from the temporal attention mechanism is fed as input into the subsequent spatial attention computation. Such an approach facilitates the establishment of temporal-spatial correlations across different time steps and channels, offering advantages for analyzing asynchronous time series with time-delay correlations. Moreover, incorporating a multi-scale strategy allows the model to discern information across different resolutions, and the innovative use of the Averaged Window Reconstruction (AWR) technique not only stabilizes the model’s reconstruction but also augments its ability to learn local data features thoroughly. Tests on the simulation dataset validate the efficacy of the proposed model in detecting accident conditions generated by the simulator. Further experiments on real nuclear power data demonstrate the effectiveness of the proposed model in capturing more extensive temporal-spatial correlations within the asynchronous MTS data, as evidenced by its superior anomaly detection performance. It can detect the occurrence of anomalous events at an earlier stage, which is crucial for early warning and prompt intervention in case of faults.

## 2. Related Works

As a pivotal component of condition-based maintenance for NPPs, automatic anomaly detection techniques are employed to assist the operator in promptly evaluating the current operational status and providing vital information for subsequent decision-making and operations. They are crucial for ensuring the safe and economical operation of NPPs. Anomaly detection methods applied to the process monitoring of NPPs can be mainly categorized into model-based or data-driven approaches. Model-based methods offer the advantage of seamlessly integrating mechanistic knowledge, thus providing strong interpretability. However, they are constrained by the requirement for comprehensive prior knowledge. Additionally, modeling complex systems poses significant challenges, and assumptions made to simplify modeling may significantly deviate from real-world conditions.

With the advancement of I&C and the industrial Internet, data-driven approaches have demonstrated enormous potential for achieving intelligent and automatic monitoring in NPPs. In particular, methods based on deep learning have emerged as a hot research topic in the current landscape. Deep learning techniques have demonstrated remarkable potential in extracting feature representations from complex nonlinear datasets, including high-dimensional MTS data [22].

The process monitoring of NPPs could be considered an issue of anomaly detection for MTS data to some extent. Among previous research on deep learning-based anomaly detection approaches, some methods primarily focus on leveraging the coupling relationships between variables to achieve anomaly detection of MTS. The essence of GDN [12] lies in learning the relationships between sensors and detecting deviations from normal patterns. This method capitalizes on the complex inter-sensor relationships by employing a graph-based attention mechanism. It establishes dependencies between sensors through embedding representations and a learned graph structure and identifies and explains anomalies via graph deviation scoring. DAGMM [13] is an unsupervised anomaly detection method that integrates a deep autoencoder with a Gaussian Mixture Model (GMM). It employs the deep autoencoder for feature extraction to obtain a compressed representation, which is then merged with the reconstruction error to form a composite feature vector. This composite feature vector is utilized by the Gaussian Mixture Model to perform anomaly scoring based on probabilistic distributions. Somehow, these kinds of methods neglect the sequential associations over time or only preserve very limited local temporal correlations through mechanisms such as sliding windows.

In contrast, another category of methods places a stronger emphasis on the importance of learning temporal dependencies. This is achieved by leveraging networks with sequential modeling capabilities to capture the contextual dynamics over time. As the most widely applied solutions for sequential modeling tasks, Recurrent Neural Networks can process variable-length time-series inputs and capture contextual temporal dynamics. RNN-based models such as LSTM-NDT [15] use Long-Short-Term Memory (LSTM) to predict data of the next timestamp. The prediction residuals are evaluated with a non-parametric dynamic thresholding approach to detect anomalies. The LSTM-VAE [16] framework effectively projects multimodal observations along with their temporal dependencies into a latent space, utilizing a sequential arrangement of LSTM and VAE layers. This framework is adept at estimating the expected distribution of multimodal inputs from their representations in the latent space. Anomaly detection is accomplished by evaluating the log-likelihood of real-time observations against this expected distribution. TranAD [17] leverages an encoder–decoder architecture supplemented with a self-attention mechanism to effectively capture contextual temporal dependencies. Additionally, an adversarial training strategy is implemented to enhance performance. Anomalies are identified with anomaly scores based on reconstruction errors. It is well known that transformer-based models, such as TranAD, are skilled at learning global relevance representations among various timestamps by utilizing temporal attention mechanisms. However, the spatial correlations between variables may not be captured comprehensively. The means of point-wise embedding for observations at the same timestamps, combined with the temporal-only attention mechanism, may not be able to extensively capture temporal-spatial correlations within the MTS data. Therefore, these methods are not suitable for handling asynchronous MTS data with time-delay correlations.

Recently, increased attention has been paid to enhancing spatial-temporal feature extraction in various transformer-based methods. For instance, PatchTST [23] demonstrated the advantages of patch-wise embedding for time-series embedding representation. CrossFormer [24] applied a two-stage attention mechanism, including a cross-time stage and a cross-dimension stage, to achieve stronger MTS prediction capabilities. iTransformer [25], on the other hand, revised the original transformer’s mechanism by utilizing MLP to capture temporal correlations and employing self-attention mechanisms to grasp spatial correlations, resulting in commendable outcomes. These approaches have made significant advancements over the vanilla transformer in terms of capturing temporal-spatial correlations. However, they do not focus on anomaly detection tasks and lack investigation into asynchronous MTS, which is the focus of this study.

In summary, deep learning-based methods have shown significant promise in anomaly detection for MTS data, which is suitable for industrial process monitoring [26,27]. However, there has been no targeted solution for addressing the issue of strong temporal lag correlations among sensors, which commonly exist in real industrial settings like nuclear power plants. This gap potentially hinders the model’s ability to effectively establish correlations between sensors, thus impacting anomaly detection performance. Motivated by this, we attempt to capture extensive spatial correlations and temporal dependencies through a temporal-spatial transformer architecture, thereby addressing the challenge of extracting features from asynchronous MTS data.

## 3. Problem Setup

### 3.1. Reconstruction-Based Method

Nuclear power plants incorporate thousands of diverse sensors tasked with monitoring various operational parameters, thereby assessing the operational status of nuclear power units. The SCADA (Supervisory Control and Data Acquisition) system [28] collates and archives real-time measurement data from these sensors, forming an MTS dataset of NPPs’ historical operational data. Consequently, the condition monitoring of NPPs can be considered an anomaly detection task for MTS data. The MTS dataset derived from *N* sensors over a designated time sequence of length *T* can be conceptualized as matrix $D\in R^{N\times T}$. Here, each column signifies the observations collected from sensors at a distinct timestamp, whereas each row encapsulates the measurements recorded by a specific sensor throughout the given period. The matrix $D\in R^{N\times T}\;$ can be expressed as:

(1) $$D=\left|\begin{array}{cccc} x_{1,1} & x_{1,2} & \cdots & x_{1,T}\\ x_{2,1} & x_{2,2} & \cdots & x_{2,T}\\ \vdots & \vdots & \ddots & \vdots \\ x_{N,1} & x_{N,2} & \cdots & x_{N,T} \end{array}\right|,$$

where $x_{i,j}$ denotes the observation of the ith sensor at the jth sampling timestamp. Reconstruction-based unsupervised MTS anomaly detection methods primarily leverage a training set consisting of normal data to learn the data patterns and dependencies under normal operational conditions. The reconstruction for input MTS can be regarded as trying to seek a reconstruction function *f*, which can be denoted as

(2) $$f\left(x_{i.j}\right)=w^{T}\phi \left(x_{i,j}\right)+b,$$

where ϕ(·) constitutes a mapping from the input space to the reconstruction feature space, *w* signifies the weight matrix, and *b* denotes the bias term. Consequently, the reconstruction error of $x_{i,j}$ in the training set can be formulated as

(3) $$E\left(x_{i,j}\right)={\left(f\left(x_{i,j}\right)-x_{i,j}\right)}^{2},$$

and the objective of the training process is to develop a model capable of accurately reconstructing normal operational data, achieved by minimizing the total reconstruction error, which can be represented as

(4) $$E_{total}=\frac{1}{N\times T}\sum_{i=1,j=1}^{N,T}E(x_{i,j}).$$

For a well-trained reconstruction model, anomaly detection can be achieved based on the principle that normal data can be accurately reconstructed by the model, indicated by minimal reconstruction errors. Conversely, anomalous data cannot be adequately reconstructed, resulting in significantly larger reconstruction errors. By selecting an appropriate threshold for the reconstruction errors, it is feasible to discriminate between normal and abnormal states for univariate time series. However, for anomaly identification of multi-sensor systems, simply averaging the sum of the reconstruction errors across channels is inappropriate due to the implicit assumption that all variables are accorded equal weight and the neglect of inter-variable correlations. Consequently, a more suitable monitoring indicator is required to accurately reflect the system’s state. Therefore, we utilize the Mahalanobis distance [29,30] of the reconstruction errors as the monitoring indicator, since its consideration of the inter-variable covariance renders it more effective for the assessment of multi-variable system states. The Mahalanobis distance of the reconstruction errors for the jth sampling timestamp can be calculated as:

(5) $$M_{j}=\sqrt{{\left(RE_{j}-\bar{E}\right)}^{T}S^{-1}\left(RE_{j}-\bar{E}\right)},$$

where $M_{j}$ and $RE_{j}$ denote the Mahalanobis distance and reconstruction error of the jth sampling timestamp in the test set, respectively, while $\bar{E}$ and $S^{-1}$ are the mean value and the inverse covariance matrix of the reconstruction errors in the training set (comprised of normal data), respectively. Therefore, the occurrence of anomalous events can be identified by the monitoring indicator with a given threshold, which is confirmed based on the Mahalanobis distance for the reconstruction errors of the normal data.

### 3.2. Issues of Asynchronous Correlations

As previously mentioned, a specific issue that needs to be addressed in this study pertains to the challenge of time-lagged correlations among related variables of real-world nuclear power operational data. Such correlations represent a form of inter-variable coupling. However, due to various factors, their changes are not synchronized over time. This asynchronous correlation is commonly observed in actual nuclear power operational parameters, although the extent of the time lag can vary significantly. Generally speaking, the causes of asynchronous correlations can primarily be attributed to the following reasons:

(1) Differences in the locations of sensors. Sensors of various types are placed in different locations to measure corresponding physical quantities, leading to differences in response times when changes occur. For example, during the process of power ramp-up, the temperature of the coolant within the reactor core rises rapidly. Temperature sensors located in the Reactor Coolant Pump (RCP) system promptly detect this change. However, sensors situated in the auxiliary cooling system or somewhere at further distances might only register the corresponding temperature variations after a delay of several minutes or even hours. Thus, although these variables are strongly correlated due to their underlying mechanisms, there is a significant time lag in their responses.
(2) Differences in response rates among variables. For instance, the current and vibration signals of the main pump are transient variables, with changes occurring almost instantaneously during pump shutdown, whereas temperature is a gradual variable that changes slowly. This leads to the time-lagged correlations between variables with different response rates.
(3) Differences in noise levels. Different levels of noise also exert a certain influence on the correlation among variables. In nuclear power data, the noise levels associated with various variables can vary significantly. For example, signals such as vibration and flow rate often exhibit higher noise levels and greater fluctuations, while signals like temperature are almost devoid of noise. These variations in noise levels can impact the analysis of correlations between variables.

**Figure 1:**
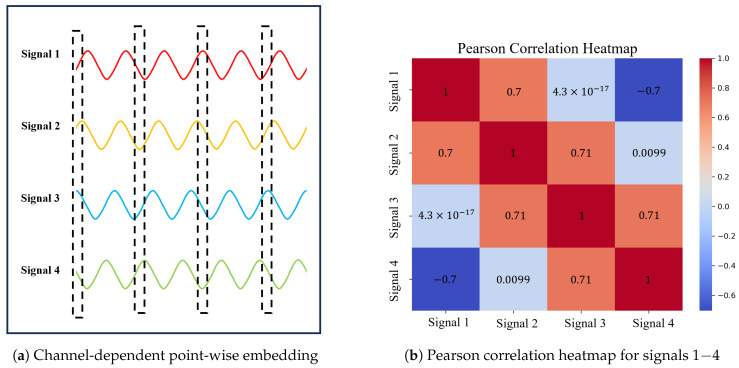
A brief schematic diagram illustrating the issues caused by time-lagged correlations for many current approaches.

Many existing deep learning-based approaches treat multidimensional observations at the same time step as a token to conduct point-wise embedding, followed by extracting features from the embedding vectors. This process restricts models to only learning synchronous cross-channel spatial correlations, rendering them incapable of capturing the asynchronous correlations mentioned earlier. As shown in Figure 1a, signals 1–4 represent four sine signals with asynchronous correlations, presenting a fixed phase difference between adjacent signals. However, when multi-dimensional observations at the same time step are treated as tokens for point-wise embedding, the resultant vector representations fail to capture these strong asynchronous correlations. This limitation is evident from the Pearson correlation heatmap in Figure 1b, where the originally strong correlations among the four signals demonstrate significant variance under this approach. Consequently, these approaches are not able to fully exploit the dependencies between variables for modeling, thereby impacting the model’s reconstruction and anomaly detection performance.

## 4. Proposed Approach

### 4.1. Anomaly Detection Strategy

**Figure 2:**
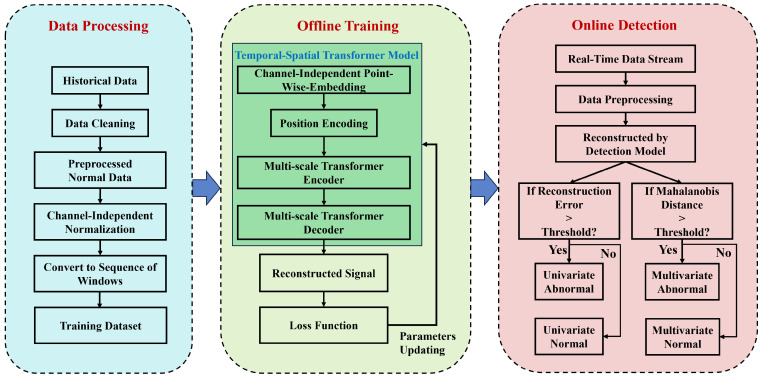
Workflow diagram of the proposed temporal-spatial transformer-based anomaly detection model.

Figure 2 illustrates the workflow of the proposed anomaly detection model based on a temporal-spatial transformer.

As shown in Figure 2, the initial step consists of data cleansing of the raw historical operational data from NPPs. This process encompasses timestamp alignment, imputation of missing values, and identification and correction of erroneous data. After that, the data under normal conditions are subjected to channel-wise normalization to mitigate the impact of amplitude level differences among various parameters. Subsequently, the MTS data are transformed into a series of sequences using a sliding-window technique, serving as the input data for model training. The training dataset, after undergoing reconstruction through the temporal-spatial transformer model, yields reconstructed signals. The widely used Mean Squared Error (MSE) loss function is employed, and the model parameters are updated through the gradient descent backpropagation process. The trained model can then distinguish abnormalities based on the principle that normal data can be accurately reconstructed, whereas abnormal data exhibit significant reconstruction errors. It is noteworthy that univariate anomalies are more aptly identified using the reconstruction error or anomaly scores from individual channels. In contrast, for anomalies in multi-sensor systems, a monitoring indicator constructed from the Mahalanobis distance of the reconstruction errors is utilized. It should be mentioned that the threshold for anomaly detection is determined based on the statistical distribution of the reconstruction errors and Mahalanobis distances in an anomaly-free validation set, following a certain criterion (such as the three-standard-deviation rule). This approach effectively distinguishes between normal and abnormal data while minimizing false positives due to the larger reconstruction errors of the normal data in the test set compared to the training set.

### 4.2. Temporal-Spatial Transformer Model

To address the above-mentioned issues of asynchronous correlations, we employed a two-stage temporal-spatial attention mechanism to capture more extensive temporal-spatial correlations between different time steps and variables.

**Figure 3:**
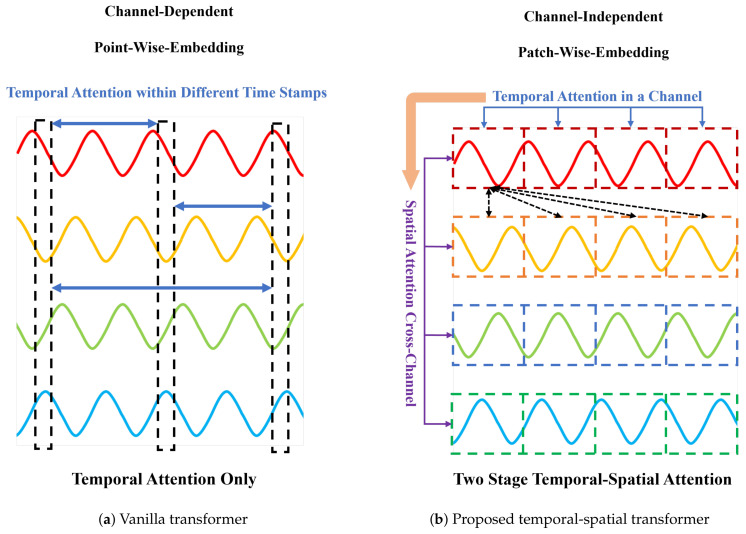
Comparison of embedding representations and attention mechanisms between the vanilla transformer and the proposed temporal-spatial transformer.

As depicted in Figure 3a, the vanilla transformer can only establish associations between tokens across different time steps through the temporal-attention mechanism. Our proposed method begins by employing a channel-independent patch-wise embedding technique for input data embedding representation, followed by applying a temporal attention mechanism to each variable along the temporal dimension. As shown in Figure 3b, the outputs thus generated serve as inputs for the subsequent stage, where spatial attention scores across channels are computed. Through the utilization of a two-stage attention mechanism, we facilitate the acquisition of more extensive temporal-spatial correlations across different time steps and variables.

**Figure 4:**
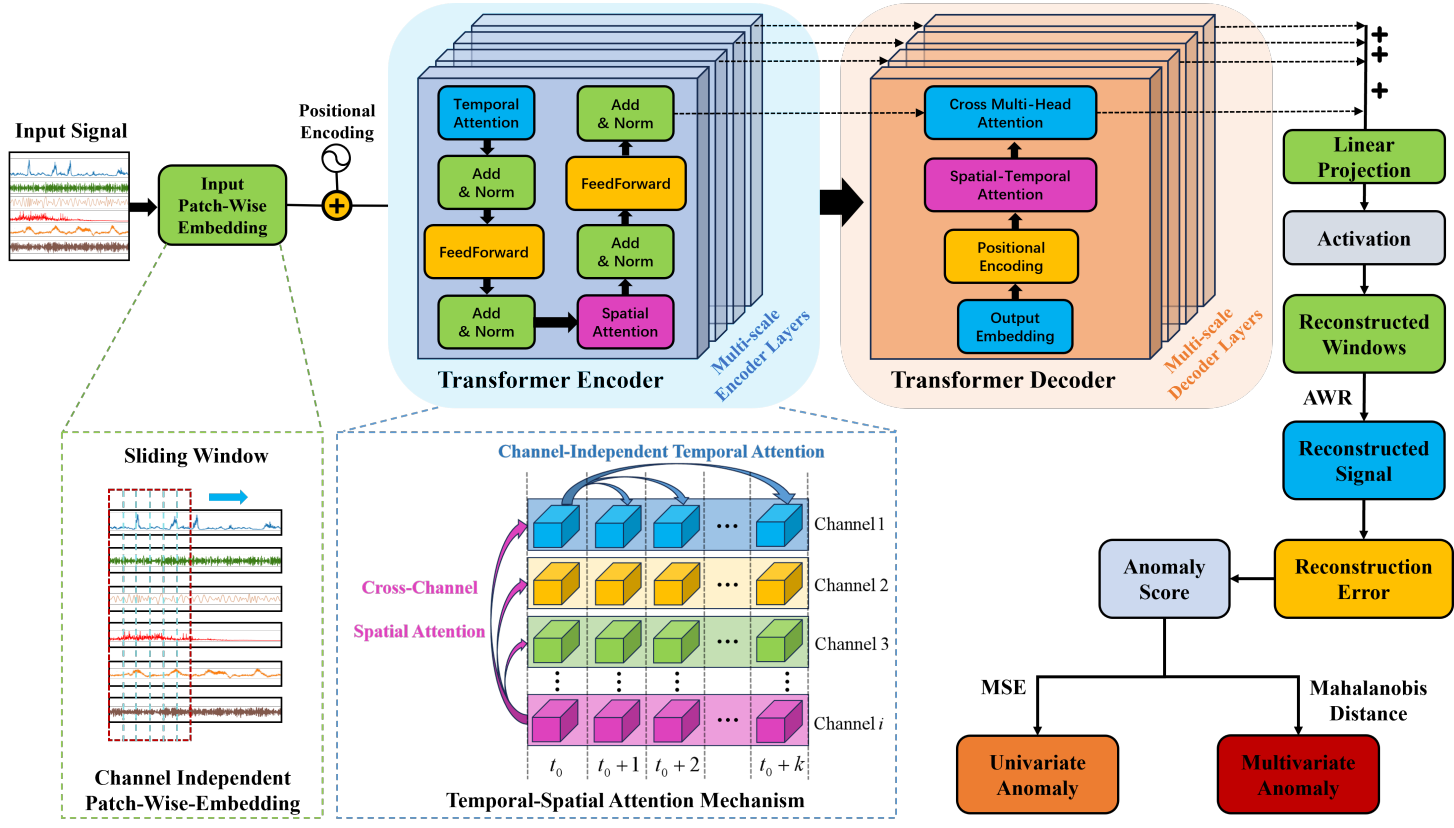
The architecture of the proposed temporal-spatial transformer anomaly detection model.

**Figure 5:**
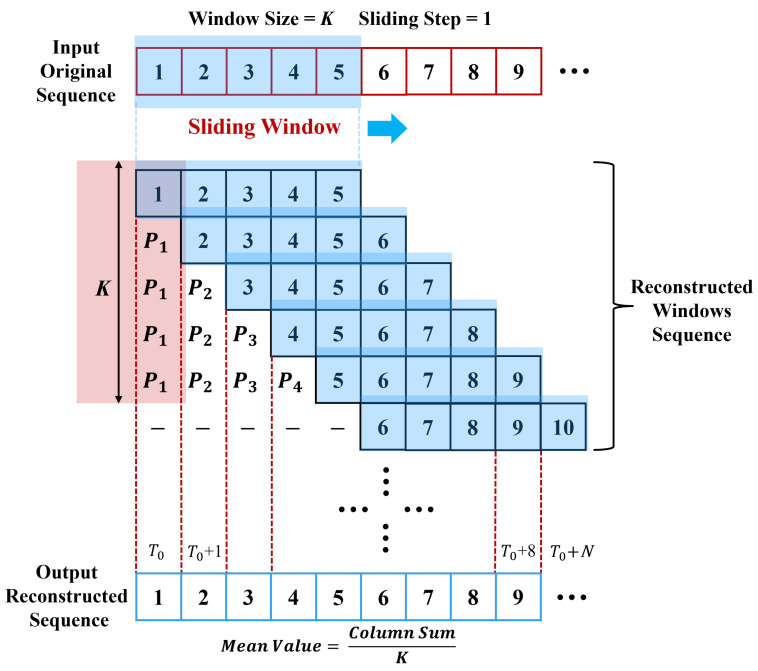
A schematic diagram of the proposed Averaged Window Reconstruction (AWR) technique.

The network architecture of the proposed temporal-spatial transformer-based anomaly detection model is illustrated in Figure 4. Similar to the vanilla transformer, the proposed temporal-spatial transformer also adopts an encoder–decoder structure to extract features and reconstruct signals. Its distinctions from the vanilla transformer model can be summarized as follows:

(1) Channel-Independent Patch-Wise Embedding. As previously mentioned, the model proposed in this article employs channel-independent patch-wise embedding instead of the traditional embedding representation method utilized in the original transformer model. By this means of embedding, the input MTS segments are embedded into a vector through a linear projection and then added with position encoding. This embedding method offers several advantages. Primarily, compared to point-wise embedding, patch-wise embedding can capture richer and more stable local association information. Furthermore, channel-independent embedding representations align better with the use of temporal attention mechanisms in the initial phase.
(2) Two-Stage Temporal-Spatial Attention Mechanism. In the vanilla transformer, the self-attention mechanism is only used to capture temporal dynamics over time, and the spatial correlations are not fully exploited. The two-stage temporal-spatial attention mechanism involves first calculating the temporal attention scores for each channel’s data, followed by using the temporally associated channel data as input for the second-stage spatial attention mechanism to establish cross-channel spatial correlations. This approach facilitates a more comprehensive capture of deep temporal-spatial associations, including the aforementioned asynchronous associations. With the help of a two-stage temporal-spatial attention mechanism, we can model the correlations among observations of different channels and time steps within the MTS data, which is key to addressing the issue of asynchronous time-delay correlations.
(3) Multi-scale feature fusion strategy. As is widely known, NPPs’ operational data encompass a diverse array of variables, such as current, temperature, flow rate, pressure, etc. Due to differences in the data characteristics of these variables, it is challenging to determine a fixed window size that can be universally applicable to all variables. Considering this, we have adopted a multi-scale feature fusion approach in our research. By employing sliding windows of varying sizes, our model is capable of extracting features at different scales. These features are then integrated to enhance the model’s performance. The use of a multi-scale mechanism allows the model to extract and integrate features of MTS data from different scales, thereby obtaining more stable multi-scale feature representations. In the process of signal reconstruction, features of various scales are fused, as shown in Figure 4.
(4) Averaged Window Reconstruction. As is widely known, the input MTS data are transformed into a multiple-window sequence as the model’s input through a sliding-window process. Therefore, the output of the temporal-spatial transformer model is still a sequence of reconstructed windows. To obtain the reconstruction signal of the original MTS data, a typical resolution involves using the mean value or a specific value of the window (the first or the last value) to represent the reconstructed signal corresponding to that time step. Here, we propose a new approach called Averaged Window Reconstruction (AWR). Utilizing AWR, the sliding step is set as 1, and the sliding window size is *K*. Therefore, every single time step is reconstructed by a series of windows of number *K*. In the proposed AWR method, we take the average of the corresponding data points from all reconstructed windows that contain a particular timestamp *t* as the reconstruction data R(t), as shown in the equation below:
  (6) $$R\left(t\right)=\frac{RW_{1}\left(t\right)+RW_{2}\left(t\right)+\cdots +RW_{n}\left(t\right)}{n},n\in \left[1,K\right],$$
  where $RW_{n}\left(t\right)$ denotes the reconstruction value of timestamp *t* from the *n*th reconstructed window. In terms of computational complexity, the proposed AWR approach is nearly equivalent to using the mean value of a single reconstructed window as the reconstructed data point; therefore, it will not result in a decrease in efficiency. Furthermore, in the context of real-time data-stream processing, the data point for a given time instance *t* is determined by the *n* reconstruction windows, n∈[1,K]. The reconstruction value for time *t* is updated as the window slides, rather than providing the reconstruction value for *t* after all *K* windows have been reconstructed. This is done to meet the response time requirements for real-time monitoring. Thus, the proposed AWR approach can achieve more stable reconstruction and better local feature capture without increasing the computational complexity or response time. Notably, at the start and end of the MTS, the average of existing reconstruction values is utilized for padding. A detailed schematic is illustrated in Figure 5.

## 5. Experiments and Results Discussion

This section primarily encompasses two categories of experiments: one involves experiments conducted on a public simulation dataset [31] generated by the PCTRAN simulation software [32], and the other involves experiments conducted on a dataset of a specific fault case derived from real nuclear power plant data. This design is motivated by the fact that, on the one hand, severe accident conditions are extremely rare in real-world NPP operations, while typical accident conditions can be generated by the PCTRAN simulator. On the other hand, the data characteristics of real operational data may differ from simulated data to some extent, such as a higher noise level. Therefore, conducting experiments on both a simulation dataset and a real NPP dataset is necessary for validating the model’s effectiveness in detecting typical accident conditions and handling asynchronous MTS anomaly detection tasks.

### 5.1. Experiments on Simulation Dataset

#### 5.1.1. Simulation Dataset and Metrics

The simulation dataset employed in this work is an open dataset generated by the Personal Computer Transient Analysis simulator (PCTRAN, developed by the Micro-simulation Technology Corporation, Montville, NJ, USA. ), which is a PC-based simulator that is widely used to simulate various kinds of accident and transient conditions in NPPs. The simulation dataset used in this experiment encompasses six types of accident conditions and normal conditions of a pressurized water reactor nuclear power plant. The six types of accident conditions include Loss of Coolant Accident in Hot Leg (LOCA), Loss of Coolant Accident in Cold Leg (LOCAC), Load Rejection (LR), Steam Generator Tube Rupture (SGTR), Steam Line Break Inside Containment (SLBIC), and Steam Line Break Outside Containment (SLBOC). Each accident condition dataset spans 300 s, with a sampling interval of 1 s. The dataset comprises 70 variables reflecting the operational status of NPPs, including various kinds of signals, such as temperature, flow rate, pressure, liquid level, and power. The initiation time for the accident conditions is uniformly set at the 150th second. The statistics of the simulation dataset are presented in Table 1.

To verify the anomaly detection performance of our approach, comparative experiments were carried out between the proposed temporal-spatial transformer model and four other representative models: DAGMM [13], LSTM-VAE [16], TranAD [17], and PCA [33]. To compare the performance of the above-mentioned anomaly detection models quantitatively, we utilized precision, recall, and the F1 score as the evaluation metrics. The calculation formula for each metric is as follows:

(7) $$P=\frac{TP}{TP+FP}\;\;,$$

(8) $$R=\frac{TP}{TP+FN}\;\;,$$

(9) $$F1=\frac{2\times P\times R}{P+R}\;\;.$$

Precision measures the true-positive rate, with higher precision implying a lower false-alarm rate. Conversely, recall quantifies the fraction of accurately detected anomalies out of all abnormal samples, with higher recall translating to a lower missed-alarm rate. The F1 score, a commonly utilized metric for assessing model performance, offers a balanced evaluation of precision and recall.

#### 5.1.2. Results and Analysis

**Figure 6:**
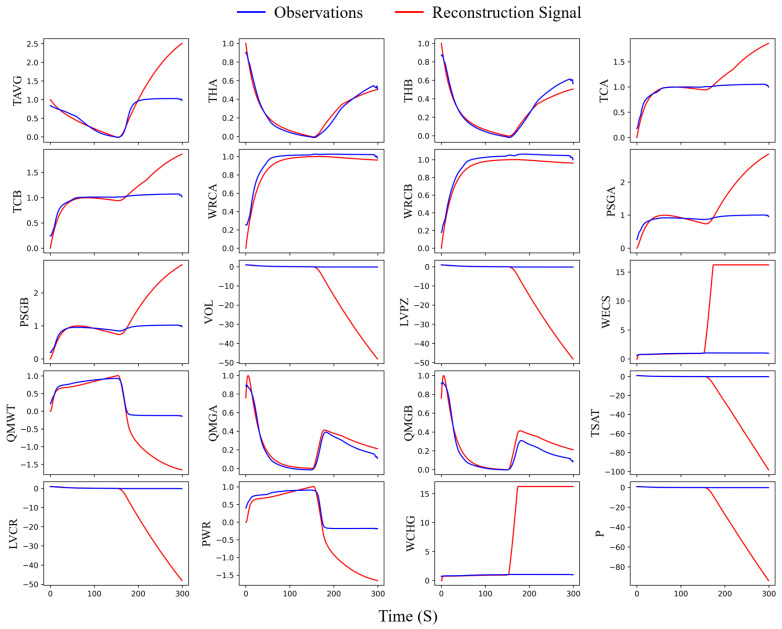
Experimental results of temporal-spatial transformer model on LOCAC accident condition dataset. (horizontal axis: time index; vertical axis: normalized observations. The abbreviations for the variable names in the figure can be cross-referenced with the Table A1 in Appendix A to ascertain the corresponding variables).

**Figure 7:**
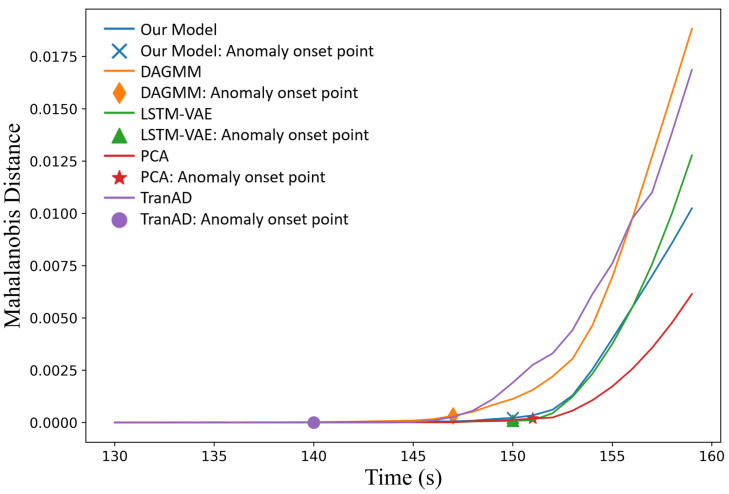
Mahalanobis distance of reconstruction errors across different models on the LOCAC accident condition dataset (horizontal axis: time index; vertical axis: normalized Mahalanobis distance).

The models utilized for comparative experiments were trained using data under normal conditions and subsequently validated on the six different accident datasets to assess their detection performance. Figure 6 presents a comparison of the reconstructed signals with the original signals for the temporal-spatial transformer model on the LOCAC accident dataset. It can be seen that nearly all signals could be accurately reconstructed with minimal reconstruction errors before the fault insertion. However, after the fault is introduced (after 150 s), the reconstruction error for some variables related to the fault condition significantly increases, whereas for others unaffected by the fault condition, the error remains nearly unchanged. Given that all six accident conditions represent multivariate severe faults, the Mahalanobis distance of the reconstruction errors serves as the monitoring indicator. Figure 7 illustrates the Mahalanobis distances of the reconstruction errors for the different models in response to the LOCAC accident conditions.

It should be noted that the threshold for anomaly identification was determined based on the statistical distribution of the Mahalanobis distance of the reconstruction errors observed in the validation set. The anomaly onset point indicates the start of an anomalous event identified by each model. As shown in Figure 7, LSTM-VAE and our model detected anomalies at the timestamp that the fault condition was introduced, whereas PCA identified the anomaly at the subsequent timestamp. This immediate detection can be attributed to the significant data variations caused by the inserted fault condition. Additionally, the three best-performing models exhibited relatively low reconstruction errors on the normal data in the test set. In contrast, TranAD and DAGMM generated some false alarms due to the reconstruction errors of the normal data in the test set exceeding the predefined thresholds. Although the localization of the anomaly onset points was dependent on the choice of thresholds to some extent, it can be seen from the trend curves that the inflection point of the proposed model was located at an earlier timestamp compared to the best-performing methods. This characteristic is helpful for the early detection of anomalous events. The evaluation metrics of all models on the six-accident-condition dataset are shown in Table 2. To mitigate the effects of model instability, the final metrics listed in Table 2 represent the average values of the monitoring indicators obtained from five independent training sessions of each model.

As depicted in Table 2, the proposed TS-Trans model achieved superior or comparable detection performance across all six fault datasets, validating the efficacy of the proposed model in detecting anomalies generated by the simulator. However, the performances of the different models exhibited minimal discrepancies, and the simulation data exhibited few asynchronous correlations. To validate the proposed method’s advantages in detecting anomalies in asynchronous MTS data, more comparative experiments were conducted on the real NPP operational data.

### 5.2. Experiments on Real NPP Dataset

It is widely acknowledged that real nuclear power data can exhibit distinct characteristics compared to simulated data, which may attributed to noise and disturbances inherent in actual operational environments, compounded by discrepancies in the response times across various parameters. Therefore, to validate the performance of anomaly detection models, we need a dataset comprised of real-world operational data.

#### 5.2.1. Real NPP Dataset

As a safety-critical system, operational data under accident conditions are extremely rare, as the vast majority of operational data are under normal conditions. Therefore, we select data from a real instance of the main pump experiencing an emergency shutdown to validate the effectiveness of the proposed model in capturing time-delayed correlations and achieving anomaly detection for asynchronous MTS data.

The real NPP dataset is composed of historical observations from 30 types of sensors, including temperature, current, flow rate, and vibration signals. It should be noted that all the observations of the variables have undergone preprocessing, resulting in a consistent sequence length and synchronized timestamps across the dataset, with a time interval of 1 s. Consequently, the dataset composed of real observational values can be represented as a matrix of multidimensional time series. The real NPP dataset includes a pump trip anomaly, with the anomalous segments being manually annotated to enable a quantitative assessment of the anomaly detection performance. It is important to highlight that the raw data from related sensors inherently exhibit asynchronous correlations with seconds-level time lag. However, to extensively demonstrate the advantages of the proposed method in capturing temporal-spatial correlations, we intentionally exaggerated this time lag by lagging the data of 10 variables by approximately 200 s. It should be noted that this time lag is consistently applied to both the training and testing datasets to simulate a more realistic scenario. The statistics of the real NPP dataset are presented in Table 3.

#### 5.2.2. Results and Discussion

**Figure 8:**
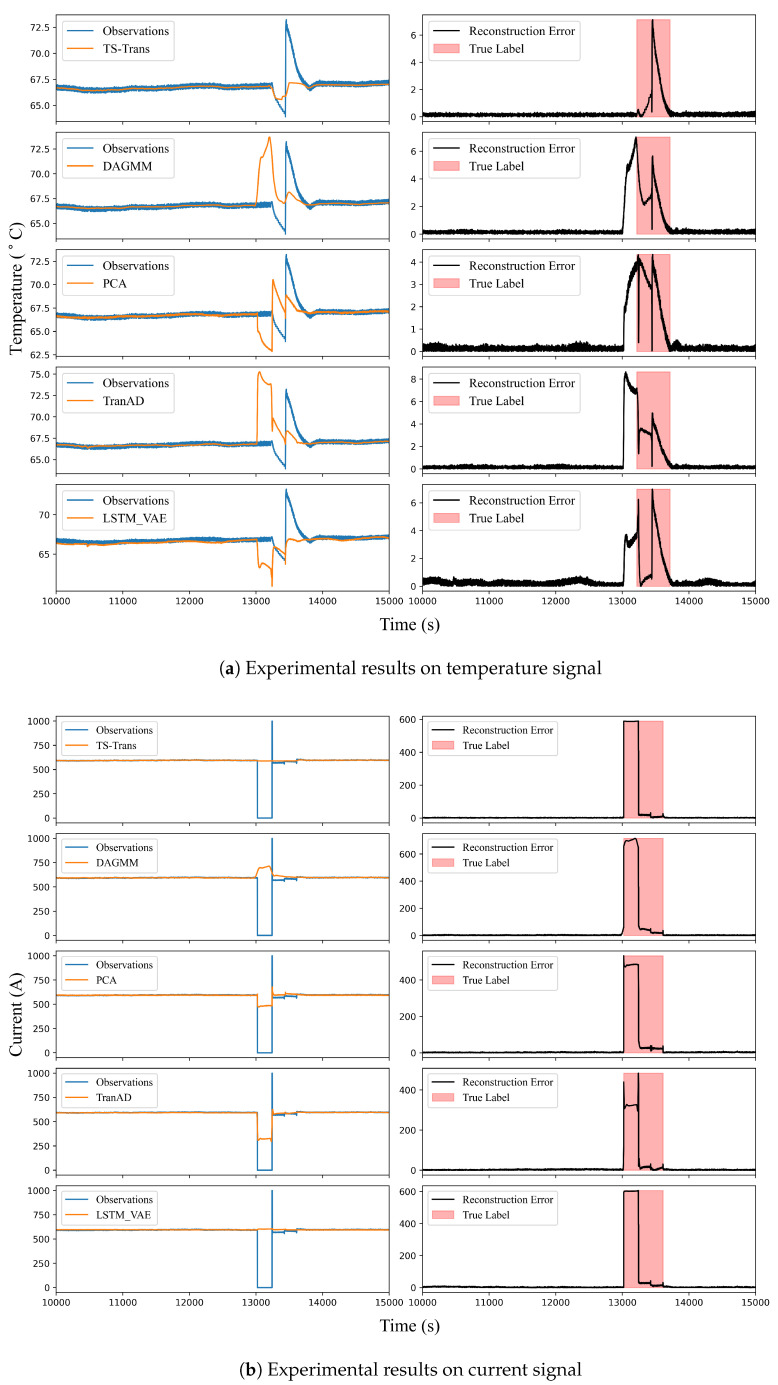
Comparison of univariate anomaly detection performance on the real NPP datasets.

The real nuclear power plant dataset, constituted by the historical operational data from sensors associated with the main pump, was divided into training, validation, and testing sets in a ratio of 5:2:3. The training and validation sets did not contain any abnormal operational conditions, whereas the testing set encompassed data from abnormal pump shutdown periods. All five models utilized for comparative experiments were trained, tested, and validated on this dataset. Given that the dataset primarily comprised steady-state operational data, segments with anomalies were relatively easy to label, allowing for manual annotation of data across each channel. Figure 8 showcases the comparative results of the reconstructed signals and residual errors of the five models. For visual comparison, a temperature signal and a current signal, without the inserted time delay of 200 s, were selected. In Figure 8, the left portion illustrates the comparison between the reconstructed signals and the actual signals of the various models, and the right portion displays the corresponding models’ reconstruction errors. Additionally, the actual anomaly labels for these signals were annotated to compare the models’ detection outcomes with the actual abnormal conditions. As depicted in Figure 8a, it can be seen that the anomalous areas with significant reconstruction errors identified by our model essentially coincide with the actual intervals of the anomalies. In comparison, the anomaly onset points identified by the other models are located ahead of the true anomaly regions, leading to a certain proportion of false alarms in univariate anomaly detection. Observations of the current signal in Figure 8b indicate that the intervals of these false alarms largely coincide with the pump shutdown periods. Thus, it can be concluded that the TS-Trans model proposed in this study possesses a superior capacity to capture the temporal-spatial characteristics of asynchronous time series, resulting in more accurate detection of univariate anomalies in the temperature signal. In contrast, the other models primarily extract synchronous temporal features, which come with inadequate learning of temporal-spatial correlations within asynchronous time series, leading to more false alarms. The evaluation metrics for each dimension were averaged to serve as the overall indicator for univariate anomaly detection, and the results are presented in Table 4.

For monitoring overall system state deviations, we continued to employ the Mahalanobis distance based on the reconstruction errors as the monitoring indicator. Furthermore, the threshold was determined by examining the statistical distribution of the reconstruction errors on the validation set. This approach was necessitated by the observation that the level of reconstruction errors in the test set typically exceeded that in the training set, even for the normal data present within the test set. Additionally, the distribution of the reconstruction errors within the validation set was more closely aligned with that of the normal data in the test set. The multivariate anomaly detection performance of the models is presented in Table 5.

**Figure 9:**
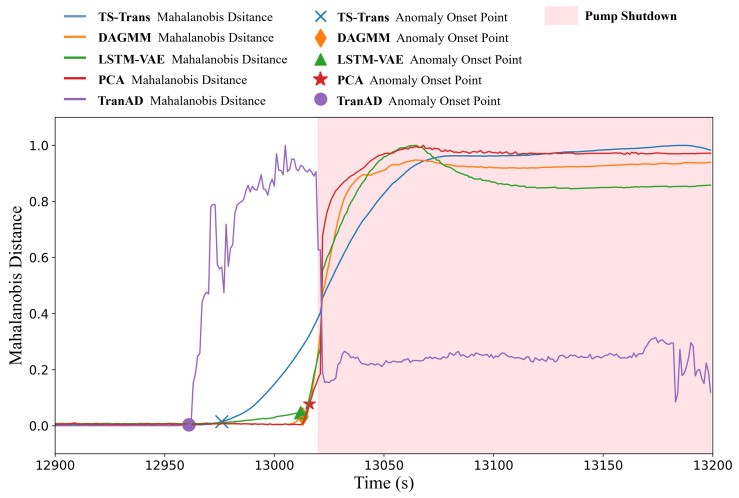
The Mahalanobis distance of reconstruction errors across different models on the real NPP dataset. (Horizontal axis: time index; vertical axis: normalized Mahalanobis distance).

As depicted in Table 5, the TS-Trans model achieved the best metrics for multivariate anomaly detection. Meanwhile, the performances of LSTM-VAE, DAGMM, and PCA were also excellent and similar to each other. TranAD exhibited suboptimal reconstruction performance on the normal data of certain variables within the test set, leading to some false negatives, which, in turn, reduced the recall of the model. The trend curves of the Mahalanobis distance based on the reconstruction errors are shown in Figure 9.

From Figure 9 combined with the evaluation metrics above, we can make the following conclusions:

- All five models successfully detected the occurrence of an anomalous event.
- The anomaly onset points identified by the models, except for TranAD, occurred after the true anomaly onset point and before the pump shutdown.
- Among these four models, the proposed TS-Trans model detected the system state deviations at an earlier stage.

In summary, the proposed temporal-spatial transformer-based anomaly detection exhibits advantages in feature extraction of asynchronous MTS data and early detection of anomalous events, which is favorable for subsequent maintenance and actions.

## 6. Conclusions

In this paper, a novel anomaly detection model based on a temporal-spatial transformer is proposed to address the challenge posed by the time-lag correlations among different sensors in real nuclear power plant scenarios. The model accomplishes the embedding representation of input data through a channel-independent patch-wise embedding approach, then harnesses a two-stage attention mechanism to capture more comprehensive temporal-spatial correlations within the MTS data. Enhanced model stability and performance are achieved through the implementation of a multi-scale strategy and the application of the averaged window reconstruction method. Experiments conducted on a simulated dataset validate the model’s effectiveness in detecting six types of accident conditions. Further experiments on a real NPP dataset, with magnified time delays, demonstrate the model’s effectiveness in detecting more complex real anomaly events, showcasing its superior capability in capturing temporal-spatial features of asynchronous MTS. Notably, this approach enables earlier detection of system status deviations, which holds significant importance for enhancing operational safety and minimizing or avoiding potential losses in NPPs.

## Figures and Tables

**Table 1:** The statistics of the simulation dataset.

Dataset	Basic Operational Status	Accident Type	Accident Severity	Total Size	Anomalies
Training Set	100% FP	Normal	/	1800	0
Test Set	100% FP	LOCA	0.01	300	150
		LOCAC	0.01	300	150
		LR	0.01	300	150
		SGTR	0.01	300	150
		SLBIC	0.01	300	150
		SLBOC	0.01	300	150

**Table 2:** Evaluation metrics of the comparison experiments on six accident condition datasets. The highest score for each metric is marked in bold.

**Model**	**LOCA**			**LOCAC**			**LR**		
	**F1**	**P**	**R**	**F1**	**P**	**R**	**F1**	**P**	**R**
DAGMM	0.983	0.966	**1**	0.966	0.934	**1**	0.977	0.955	**1**
LSTM-VAE	0.955	0.913	**1**	0.977	0.955	**1**	0.961	0.926	**1**
PCA	0.988	0.985	0.99	0.965	**0.968**	**1**	0.802	0.963	0.687
TranAD	0.982	0.965	**1**	0.956	0.916	**1**	0.966	0.934	**1**
TS-Trans	**0.997**	**1**	0.995	**0.984**	**0.968**	**1**	**0.987**	**0.974**	**1**
**Model**	**SGTR**			**SLBIC**			**SLBOC**		
	**F1**	**P**	**R**	**F1**	**P**	**R**	**F1**	**P**	**R**
DAGMM	0.946	0.898	**1**	0.977	0.955	**1**	0.983	0.966	**1**
LSTM-VAE	0.937	0.882	**1**	0.971	0.943	**1**	0.959	0.922	**1**
PCA	0.987	0.974	**1**	0.748	0.958	0.613	0.982	**0.99**	0.975
TranAD	0.966	0.934	**1**	0.922	0.855	**1**	0.974	0.95	**1**
TS-Trans	**0.993**	**0.987**	**1**	**0.984**	**0.968**	**1**	**0.988**	0.976	**1**

**Table 3:** The statistics of the real NPP dataset.

Dataset	Basic Operational Status	Dimensions	Total Size	Anomalies	Anomaly Rate
Training Set	85% FP Steady-State	30	30,000	/	/
Test Set	85% FP Steady-State	30	18,000	859	4.77%

**Table 4:** The univariate anomaly detection performance of the 5 models on the real NPP dataset. (The final evaluation metrics are taken as the mean value of the metrics from each channel, the highest score for each metric is marked in bold).

Model	Window Size	F1	P	R
DAGMM	60	0.711	0.6	0.873
LSTM-VAE	60	0.907	0.877	0.939
PCA	/	0.877	0.867	0.886
TranAD	60	0.768	0.624	**0.999**
TS-Trans	60	**0.962**	**0.928**	0.998

**Table 5:** The multivariate anomaly detection performance (based on the Mahalanobis distance of reconstruction errors) of the five models on the real NPP dataset. The highest score for each metric is marked in bold.

Model	F1	P	R
DAGMM	0.913923	0.97775	0.857918
LSTM-VAE	0.915176	0.968254	0.867615
PCA	0.920541	0.984887	0.864088
TranAD	0.854501	0.966527	0.765746
TS-Trans	**0.979798**	**0.995439**	**0.964641**

## Data Availability

Data are contained within the article.

## Appendix A

**Table A1:** Abbreviations for variables used in Figure 6.

Index	Abbreviated Name	Corresponding Variable
1	TAVG	Coolant average temperature
2	THA	Branch A hot-leg temperature
3	THB	Branch B hot-leg temperature
4	TCA	Branch A cold-leg temperature
5	TCB	Branch B cold-leg temperature
6	WRCA	Branch A coolant flow rate
7	WRCB	Branch B coolant flow rate
8	PSGA	Branch A steam generator pressure
9	PSGB	Branch B steam generator pressure
10	VOL	Coolant volume
11	LVPZ	Pressurizer liquid level
12	WECS	Emergency core cooling system flow rate
13	QMWT	Thermal power
14	QMGA	Branch A steam generator extraction thermal power
15	QMGB	Branch B steam generator extraction thermal power
16	TSAT	Pressurizer saturation temperature
17	LVCR	Core water level
18	PWR	Core thermal power
19	WCHG	Top-up flow rate
20	P	Primary side pressure of the reactor coolant system

## References

[1] Stamford L., Azapagic A. (2011). Sustainability indicators for the assessment of nuclear power. Energy.

[2] Karakosta C., Pappas C., Marinakis V., Psarras J. (2013). Renewable energy and nuclear power towards sustainable development: Characteristics and prospects. Renew. Sustain. Energy Rev..

[3] Adamantiades A., Kessides I. (2009). Nuclear power for sustainable development: Current status and future prospects. Energy Policy.

[4] Ramana M.V. (2011). Nuclear power and the public. Bull. At. Sci..

[5] Hashemian H.M. (2011). On-line monitoring applications in nuclear power plants. Prog. Nucl. Energy.

[6] Ayo-Imoru R.M., Cilliers A.C. (2018). A survey of the state of condition-based maintenance (CBM) in the nuclear power industry. Ann. Nucl. Energy.

[7] Ma J., Jiang J. (2011). Applications of fault detection and diagnosis methods in nuclear power plants: A review. Prog. Nucl. Energy.

[8] Arunthavanathan R., Khan F., Ahmed S., Imtiaz S., Rusli R. (2020). Fault detection and diagnosis in process system using artificial intelligence-based cognitive technique. Comput. Chem. Eng..

[9] Pang G., Shen C., Cao L., Hengel A.V.D. (2021). Deep learning for anomaly detection: A review. ACM Comput. Surv. CSUR.

[10] Khentout N., Magrotti G. (2023). Fault supervision of nuclear research reactor systems using artificial neural networks: A review with results. Ann. Nucl. Energy.

[11] Dong F., Chen S., Demachi K., Yoshikawa M., Seki A., Takaya S. (2023). Attention-based time series analysis for data-driven anomaly detection in nuclear power plants. Nucl. Eng. Des..

[12] Deng A., Hooi B. Graph neural network-based anomaly detection in multivariate time series. Proceedings of the AAAI Conference on Artificial Intelligence.

[13] Zong B., Song Q., Min M.R., Cheng W., Lumezanu C., Cho D., Chen H. Deep autoencoding gaussian mixture model for unsupervised anomaly detection. Proceedings of the International Conference on Learning Representations.

[14] He Y., Zhao J. (2019). Temporal convolutional networks for anomaly detection in time series. J. Phys. Conf. Ser..

[15] Hundman K., Constantinou V., Laporte C., Colwell I., Soderstrom T. Detecting spacecraft anomalies using lstms and nonparametric dynamic thresholding. Proceedings of the 24th ACM SIGKDD International Conference on Knowledge Discovery & Data Mining.

[16] Park D., Hoshi Y., Kemp C.C. (2018). A multimodal anomaly detector for robot-assisted feeding using an LSTM-based variational autoencoder. IEEE Robot. Autom. Lett..

[17] Tuli S., Casale G., Jennings N.R. (2022). Tranad: Deep transformer networks for anomaly detection in multivariate time series data. arXiv.

[18] Vaswani A., Shazeer N., Parmar N., Uszkoreit J., Jones L., Gomez A.N., Kaiser Ł., Polosukhin I. (2017). Attention is all you need. Adv. Neural Inf. Process. Syst..

[19] Islam S., Elmekki H., Elsebai A., Bentahar J., Drawel N., Rjoub G., Pedrycz W. (2023). A comprehensive survey on applications of transformers for deep learning tasks. Expert Syst. Appl..

[20] Abdulaal A., Liu Z., Lancewicki T. Practical approach to asynchronous multivariate time series anomaly detection and localization. Proceedings of the 27th ACM SIGKDD Conference on Knowledge Discovery & Data Mining.

[21] Gamboa J.C.B. (2017). Deep learning for time-series analysis. arXiv.

[22] Choi K., Yi J., Park C., Yoon S. (2021). Deep learning for anomaly detection in time-series data: Review, analysis, and guidelines. IEEE Access.

[23] Nie Y., Nguyen N.H., Sinthong P., Kalagnanam J. A Time Series is Worth 64 Words: Long-term Forecasting with Transformers. Proceedings of the Eleventh International Conference on Learning Representations.

[24] Zhang Y., Yan J. Crossformer: Transformer utilizing cross-dimension dependency for multivariate time series forecasting. Proceedings of the Eleventh International Conference on Learning Representations.

[25] Liu Y., Hu T., Zhang H., Wu H., Wang S., Ma L., Long M. (2023). itransformer: Inverted transformers are effective for time series forecasting. arXiv.

[26] Yang C., Cai B.P., Wu Q.B., Wang C.Y.S., Ge W.F., Hu Z.M., Zhu W., Zhang L., Wang L.T. (2023). Digital twin-driven fault diagnosis method for composite faults by combining virtual and real data. J. Ind. Inf. Integr..

[27] Song Q., Wang M.S., Lai W.X., Zhao S.F. (2022). On Bayesian optimization-based residual CNN for estimation of inter-turn short circuit fault in PMSM. IEEE Trans. Power Electron..

[28] Nechibvute A., Mudzingwa C. (2013). Wireless sensor networks for scada and industrial control systems. Int. J. Eng. Technol..

[29] McLachlan G.J. (1999). Mahalanobis distance. Resonance.

[30] De Maesschalck R., Jouan-Rimbaud D., Massart D.L. (2000). The mahalanobis distance. Chemom. Intell. Lab. Syst..

[31] Qi B., Xiao X., Liang J., Po L.-C.C., Zhang L., Tong J. (2022). An open time-series simulated dataset covering various accidents for nuclear power plants. Sci. Data.

[32] Cheng Y.-H., Shih C., Chiang S.-C., Weng T.-L. (2012). Introducing PCTRAN as an evaluation tool for nuclear power plant emergency responses. Ann. Nucl. Energy.

[33] Jin Y., Qiu C., Sun L., Peng X., Zhou J. Anomaly detection in time series via robust PCA. Proceedings of the 2017 2nd IEEE International Conference on Intelligent Transportation Engineering (ICITE).

